# A cascading learning method with SegFormer for radiographic measurement of periodontal bone loss

**DOI:** 10.1186/s12903-024-04079-y

**Published:** 2024-03-11

**Authors:** Hanwen Yu, Xin Ye, Wanjing Hong, Rui Shi, Yi Ding, Chengcheng Liu

**Affiliations:** 1 School of Resources and Environment, University of Electronic Science and Technology, Chengdu, Sichuan 610097 China; 2 State Key Laboratory of Oral Diseases & National Center for Stomatology & National Clinical Research Center for Oral Diseases, West China Hospital of Stomatology, Sichuan University, Chengdu, Sichuan 610041 China

**Keywords:** Periodontitis, Alveolar bone resorption, Tooth position recognition, Deep learning, Segmentation

## Abstract

**Objective:**

Marginal alveolar bone loss is one of the key features of periodontitis and can be observed via panoramic radiographs. This study aimed to establish a cascading learning method with deep learning (DL) for precise radiographic bone loss (RBL) measurements at specific tooth positions.

**Materials and methods:**

Through the design of two tasks for tooth position recognition and tooth semantic segmentation using the SegFormer model, specific tooth’s crown, intrabony portion, and suprabony portion of the roots were obtained. The RBL was subsequently measured by length through these three areas using the principal component analysis (PCA) principal axis.

**Results:**

The average intersection over union (IoU) for the tooth position recognition task was 0.8906, with an F1-score of 0.9338. The average IoU for the tooth semantic segmentation task was 0.8465, with an F1-score of 0.9138. When the two tasks were combined, the average IoU was 0.7889, with an F1-score of 0.8674. The correlation coefficient between the RBL prediction results based on the PCA principal axis and the clinicians’ measurements exceeded 0.85. Compared to those of the other two methods, the average precision of the predicted RBL was 0.7722, the average sensitivity was 0.7416, and the average F1-score was 0.7444.

**Conclusions:**

The method for predicting RBL using DL and PCA produced promising results, offering rapid and reliable auxiliary information for future periodontal disease diagnosis.

**Clinical relevance:**

Precise RBL measurements are important for periodontal diagnosis. The proposed RBL-SF can measure RBL at specific tooth positions and assign the bone loss stage. The ability of the RBL-SF to measure RBL at specific tooth positions can guide clinicians to a certain extent in the accurate diagnosis of periodontitis.

## Introduction

Periodontitis is a chronic inflammatory condition of tooth-supporting tissues that leads to destruction of the periodontal ligament and alveolar bone [1]. Periodontitis is the seventh most prevalent disease worldwide, affecting 1.09 billion people worldwide [2]. Severe periodontitis affects approximately 11.2% of the global population, and for individuals, it represents a substantial burden, both in terms of health and finances, significantly compromising their quality of life [3]. The 2018 periodontitis staging and grading classification aims to assess the severity, extent, progression rate, and response to standard therapy in patients with periodontitis. Alveolar bone loss is the main clinical feature of periodontitis, and two-dimensional intraoral radiography is the primary screening technique used for evaluating alveolar bone loss [4–6]. Rapid and accurate determination of the maximum radiographic bone loss (RBL) and calculation of the RBL (% of root length ×100) divided by age are critical for staging and grading periodontitis [7]. However, this approach is time-consuming and laborious for clinicians, making it difficult to guarantee consistency because clinicians need to correctly identify anatomic markers, including the distance from the cemento-enamel junction (CEJ) to the alveolar bone crest (ABC), to locate the physiologically healthy ridge level, the basis of bone loss, and the apical point (AP), on radiographs.

Artificial intelligence (AI) aims to reproduce human cognitive processes and can achieve the same results as clinicians in less time. Moreover, this approach excels at helping clinicians by automating time-consuming tasks. AI can improve visual diagnosis in radiology, resulting in lower error rates than human observers, opening up an exciting era of clinical and research capabilities. The detection and classification of lesions, automatic image segmentation, data analysis, extraction of radiological features, and conversion to automatic printout are significant technological developments in the application of computer-aided medicine [8]. Deep learning (DL) networks may be a useful tool for improving the accuracy and efficiency of evaluating RBL. Several studies have used DL to measure alveolar bone levels on panoramic or periapical radiographs [9–12]. However, many of these studies prioritize classifying the severity of RBL over pinpointing its exact value. Some techniques that aim to quantify RBL precisely are deemed overly complex. Moreover, many studies fail to determine the RBL for specific tooth positions, a limitation that could compromise the accurate diagnosis of periodontal disease.

Convolutional neural networks (CNNs) are the predominant DL architecture within dentistry [13]. However, their limited receptive field can hinder segmentation performance when applied to large-scale medical images. In contrast, transformer architectures excel in grasping global dependencies, meaning that they can more effectively understand long-distance dependencies and intricate structures, making them ideal for processing large images. The self-attention mechanism in transformers allows the model to adaptively determine the receptive field, enhancing the capture of features and structures across different scales and improving segmentation accuracy. SegFormer is an efficient semantic segmentation framework that integrates multiple transformer encoders to capture multi-scale features and employs a lightweight multilayer perceptron (MLP) for decoding [14]. The transformer architecture within SegFormer can adaptively determine the receptive field to handle large-scale radiographs. Furthermore, its multi-scale structure is useful for discerning hierarchical information in radiographs, such as the varying sizes of teeth and their respective components in panoramic radiographs. Additionally, the decoder’s reduced parameter count potentially mitigates the risk of overfitting in medical image segmentation. Therefore, this study focused on tooth position recognition and RBL measurement using an innovative SegFormer-based model, RBL-SF, and compare its performance with clinician evaluations on panoramic radiographs.

## Materials and methods

### Radiographic data collection

This study was approved by the Ethics Committee of the State Key Laboratory of Oral Diseases, West China Hospital of Stomatology, Sichuan University (WCHSIRB-D-2023-370), and conducted in accordance with the checklist for AI in dental research [15]. Each standardized radiograph was captured by a panoramic X-ray unit (Veraviewepocs, X550 EX-2). Digital images were captured using a charge-coupled device (CCD) sensor with a size of #1 or #2. Radiographs that were not in the standard format were excluded from the study.

### Alveolar bone level measurement

**Fig. 1:**
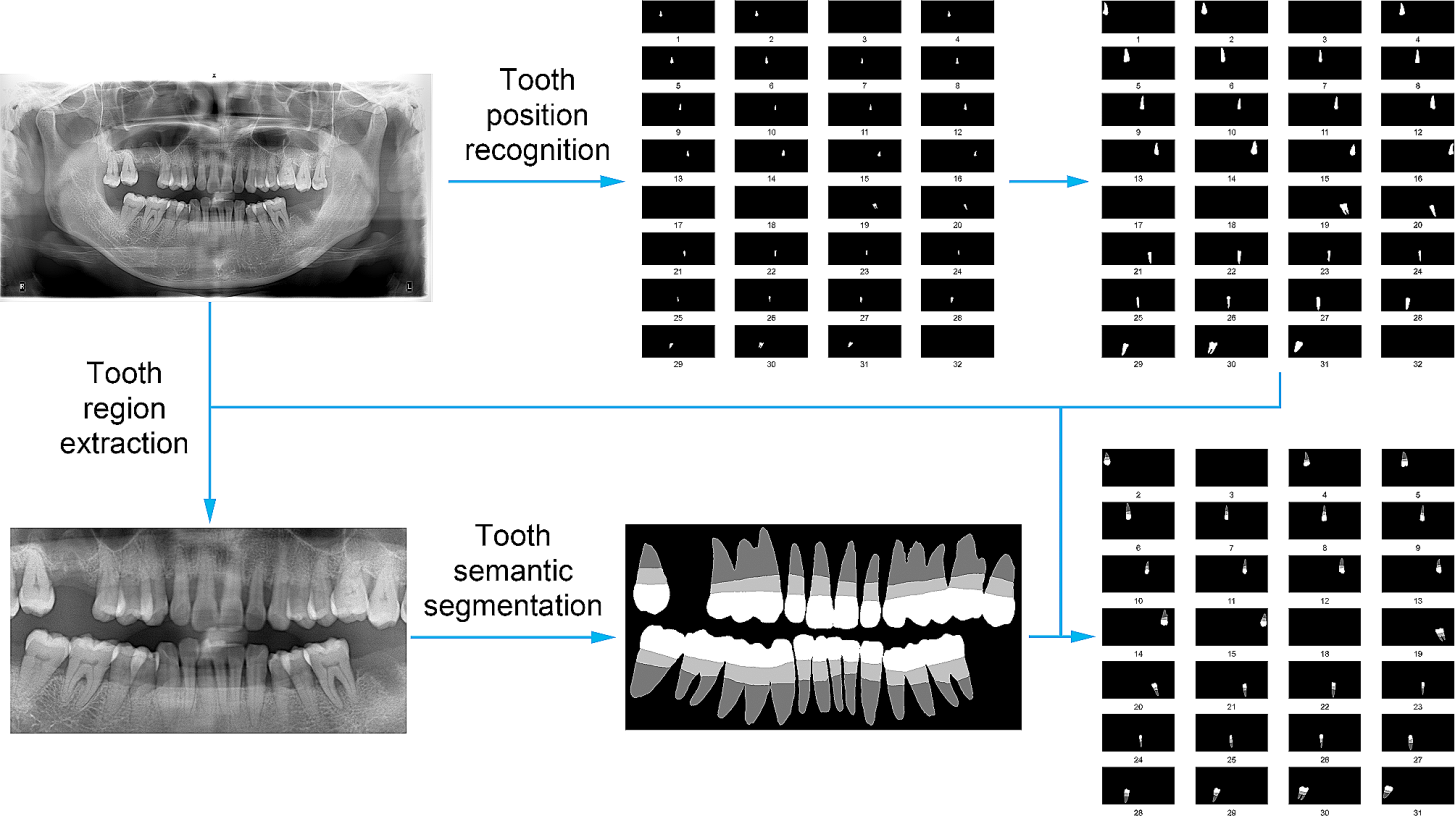
Flowchart of tooth position recognition and tooth semantic segmentation. The workflow begins with the utilization of SegFormer for tooth position recognition on panoramic images to identify the locations of 32 teeth. Based on the tooth position data, specific regions containing teeth were extracted from the panoramic radiographs. After this extraction, semantic segmentation of the teeth was performed on the cropped regions. Ultimately, combining the tooth position information with the semantic segmentation results yielded the semantic segmentation of individual teeth. The direction of the blue arrow indicates the process sequence

The teeth were numbered from 1 to 32 on the panoramic images using the universal numbering system. The objective of tooth semantic segmentation was to distinguish the crown, suprabony root part, and intrabony root section. As illustrated in Fig. 1, the process began with tooth position recognition based on the SegFormer framework. The dental regions were subsequently extracted according to the identified tooth position data, followed by tooth segmentation within the extracted dental areas. This approach significantly reduced the input image size for the tooth semantic segmentation task, allowing improved segmentation accuracy even when memory resources were constrained. Combining these results allowed the delineation of details such as the tooth crown, suprabony root part, and intrabony root section.

**Fig. 2:**
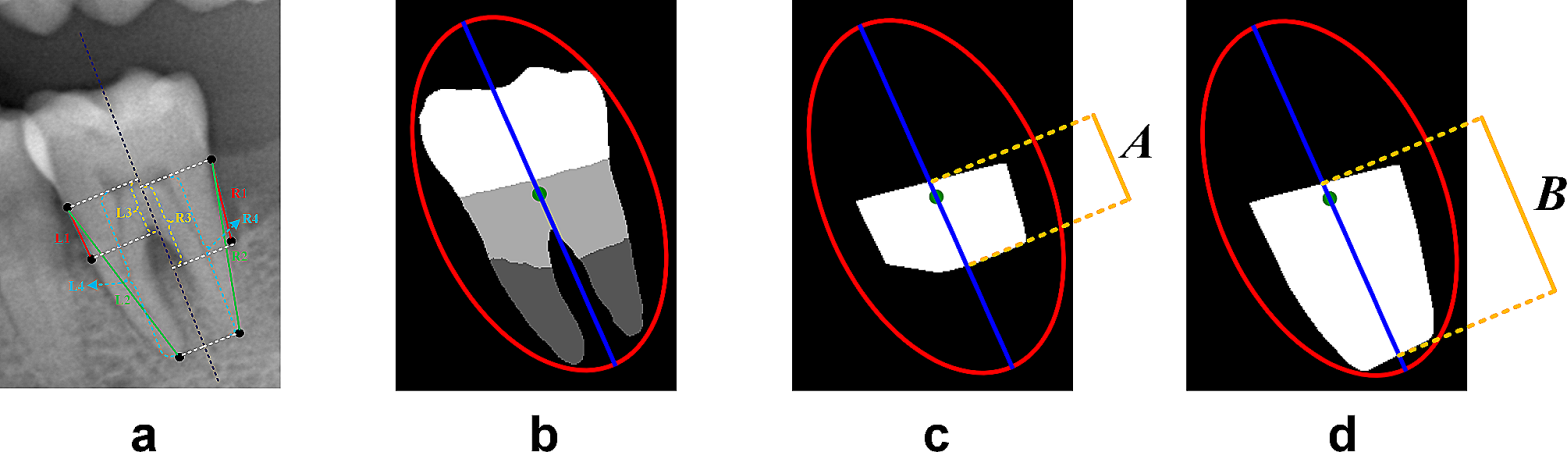
Manual X-ray bone loss measurement method and PCA measurement method based on tooth semantic segmentation results. (**a**) Two manual methods for RBL measurement are described as max (L1/L2, R1/R2) ×100 and max (L3/L4, R3/R4) ×100. The red lines indicate the length of the CEL to the ABC, and the green lines indicate the length of the CEJ to the AP. The yellow lines indicate the distance from the CEL to the ABC, and the blue lines indicate the distance from the CEJ to the AP. L and R represent proximal and distal, respectively. (**b**) The external ellipse of the tooth body and its principal axis were obtained via PCA. The red line indicates the tooth’s smallest enclosing ellipse, and the dark blue line represents the tooth’s longitudinal axis. The green dot represents the center of the red ellipse. (**c**) Length A of the principal axis of the ellipse passed through the convex hull of the suprabony portion of the root. (**d**) Length B of the principal axis of the ellipse passed through the convex hull of both the suprabony and intrabony portions of the root. PCA, principal component analysis; CEJ, cemento-enamel junction; ABC, alveolar bone crest; AP, apical point

The RBL of each tooth was calculated separately using two methods (Fig. 2a). The first used the formula max (L1/L2, R1/R2) ×100, which represents the maximum length of the proximal and distal CEL to ABC lines length as a percentage of the CEJ to AP line length. For the second method, RBL was expressed as the maximum percentage of the distance from the proximal and distal CEJ to the ABC level and the distance from the CEJ to the AP, described as max (L3/L4, R3/R4) ×100. Measuring the RBL required identifying the tooth structure’s main axis. However, due to the intricacy of the geometric projection of teeth and its deviation from traditional Euclidean geometry, conventional graphical algorithms struggle to define this main axis. Therefore, this research initially applied the circumscribed ellipse of teeth for Euclideanization, a procedure accomplished utilizing principal component analysis (PCA), as illustrated in Fig. 2b. The PCA approach involved treating the figure as a scatter plot, calculating the covariance matrix, and subsequently computing its eigenvalues and eigenvector, leading to the identification of the ellipse’s principal axis based on the maximum eigenvalue’s corresponding eigenvector and the minor axis based on the minimum eigenvalue’s corresponding eigenvector. The principal axis signifies the direction of the maximum variance in the data, indicating a broader spread in that direction. Therefore, the PCA principal axis could be equated with the tooth structure’s main axis. For RBL measurements, the PCA principal axis length within the segmented region was adequate. However, in cases with multiple tooth roots, the principal axis might traverse non-target areas, necessitating a preliminary convex hull calculation. The length of the principal axis inside the convex hull was subsequently determined, as detailed in Fig. 2c and d.

To summarize, the RBL calculation for a specific tooth position involved the following steps: a). The SegFormer tool was used to pinpoint the tooth’s position, and based on these data, the dental areas were extracted. b). SegFormer was utilized to execute semantic segmentation on the extracted panoramic images. The crown, intrabony root section, and suprabony portion of all the teeth were identified. c). The tooth position data were merged with region information to generate a bone level segmentation image for a specific tooth position. d). PCA was implemented to determine the tooth’s smallest enclosing ellipse. The principal axis of the PCA represents the tooth’s longitudinal axis. First, we calculated the length (A) of this principal axis within the convex hull of the suprabony region (Fig. 2c). Next, we computed the length (B) of this axis within both the suprabony and intrabony regions (Fig. 2d).

The RBL percentage was calculated through a ratio calculation, expressed as follows:

1 $$RBL\% =\frac{A}{B} \times 100$$

Based on the staging and grading of periodontitis criteria published in 2018, the severity of RBL was further classified as follows: stage I (RBL < 15%), stage II (RBL ranging between 15 and 33%), and stage III (RBL extending to the middle or apical third of the root; ≥33%) [7].

### Image pre-processing and augmentation

To handle large-scale panoramic images, the model leverages SegFormer, which was built upon a transformer encoder. This approach provided access to an expansive receptive field, thus boosting segmentation accuracy. The model focused on two main tasks: recognizing tooth positions and segmenting alveolar bone levels. For annotating tooth positions, the open-source tool LabelMe was used. In contrast, Adobe Photoshop CS6 was utilized for annotating alveolar bone levels.

### SegFormer model

**Fig. 3:**
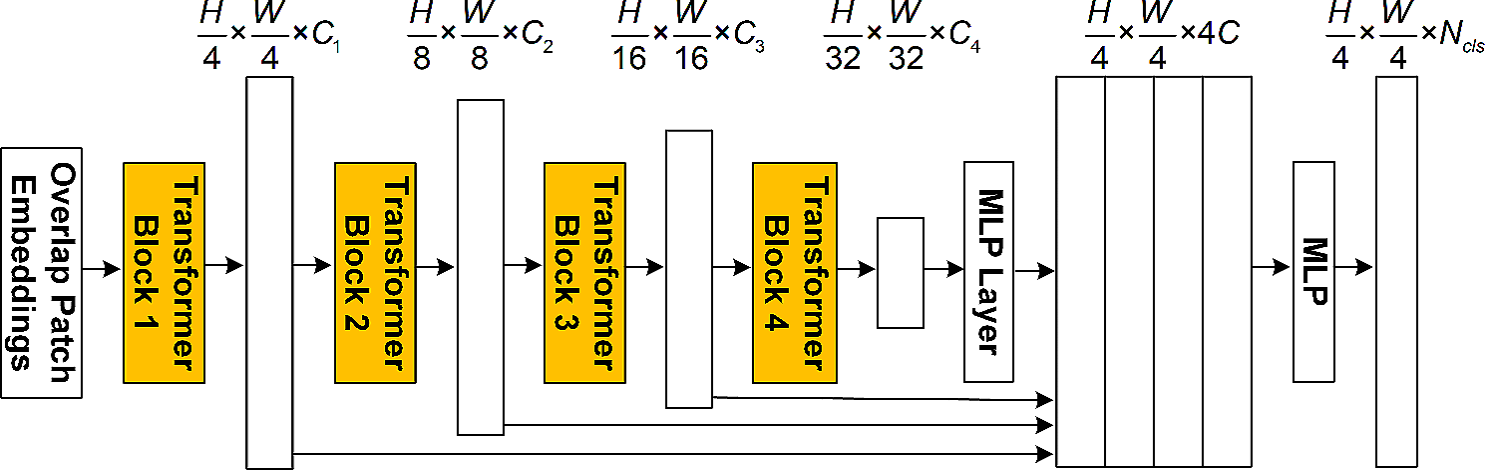
Diagram of the SegFormer model. SegFormer incorporates four transformer blocks, represented by the orange parts, with each capturing feature at scales of 1/4, 1/8, 1/16, and 1/32 of the original image dimensions. These features were unified to a consistent scale using an MLP layer and then processed through another MLP layer for classification. *H* and *W* indicate the input image’s height and width, respectively. *C*_1_ to *C*_4_ and *C* denote the feature map channel counts. *N*_*cls*_ specifies the class count. MLP, multilayer perceptron

The overall structure of SegFormer is depicted in Fig. 3. Four transformer encoding blocks are employed to derive multi-scale features, followed by the utilization of a lightweight MLP for the fusion of features at different scales, facilitating decoding and semantic segmentation. The transformer encoding modules of the SegFormer model utilized the mix transformer encoder (MiT) to effectively reduce the computational time complexity, incorporating an efficient self-attention mechanism. The MiT model included a range of models with varying parameter sizes, named MiT-B0 to MiT-B5. In this study, the moderately sized MiT-B3 model was selected as the feature extraction module for SegFormer. Once tooth position recognition was performed on the panoramic images, only the tooth region of the panoramic images was extracted for further alveolar bone level segmentation.

Different loss functions were used for the two tasks. For tooth position recognition, due to the overlap of regions among numerous teeth, a multi-label loss function was used to identify each complete tooth position, thus avoiding the restriction of assigning each pixel to only one class. In this framework, the loss function was critical for training the model to accurately predict multiple class memberships for each pixel. We defined our primary loss function, ${\mathcal{L}_{{\text{multi}}}}$, as follows:

2 $$\begin{aligned}{\mathcal{L}_{{\text{multi}}}}&=- \frac{1}{{{W_1} \times {H_1} \times {C_1}}}\\& \quad\sum\limits_{{i=1}}^{{{W_1}}} {\sum\limits_{{j=1}}^{{{H_1}}} {\sum\limits_{{k=1}}^{{{C_1}}} {\left({{y_{i,j,k}}\log ({p_{i,j,k}})+(1 - {y_{i,j,k}})\log (1 - {p_{i,j,k}})}\right)} } }\end{aligned}$$

where *W*_1_ and *H*_1_ are the width and height of the output grid, respectively. *C*_1_ was the total number of classes; for this study, there were 32 classes corresponding to different tooth positions. ${p_{i,j,k}}$represents the predicted probability that the pixel at location (*i*, *j*) belongs to class *k*, as output by the neural network after the sigmoid activation function. ${y_{i,j,k}}$was a binary indicator, which is 1 if the pixel at position (*i*, *j*) belongs to class *k* and 0 otherwise. This formulation of ${\mathcal{L}_{{\text{multi}}}}$ compels the model to predict a probability distribution over classes for each pixel that closely aligns with the true label distribution, thereby addressing the inherent challenge of multi-label segmentation wherein a single pixel could simultaneously belong to multiple classes.

In our tooth semantic segmentation model, we computed the cross-entropy loss, which assesses the difference between the predicted class probabilities and the actual labels for each pixel. For a given image with height *H*_2_ and width *W*_2_, the loss function ${\mathcal{L}_{{\text{ce}}}}$was defined as the average negative log probability of the true class across all pixels:

3 $${\mathcal{L}_{{\text{ce}}}}= - \frac{1}{{{W_2} \times {H_2}}}\sum\limits_{{i=1}}^{{{W_2}}} {\sum\limits_{{j=1}}^{{{H_2}}} {\sum\limits_{{k=1}}^{{{C_2}}} {{y_{i,j,k}}\log \left( {{p_{i,j,k}}} \right)} } }$$

where *H*_2_ and *W*_2_ are the height and width of the output grid, respectively. *C*_2_ was the number of classes, which was 4 in this segmentation task and corresponded to the four classes: background (0), intrabony root Sect. (1), suprabony root part (2), and tooth crown (3). ${y_{i,j,k}}$was a binary indicator, which is 1 if the pixel at position (*i*, *j*) belongs to class *k* and 0 otherwise. The probabilities ${p_{i,j,k}}$ were the values obtained by applying the softmax function to the network’s output logits for each pixel location (*i*, *j*) and class *k*. The softmax function converts these logits into probabilities by exponentiating each logit and then normalizing these exponentiated values across all classes for each pixel, ensuring that the probabilities for each pixel across all classes sum to 1.

### Model training and validation

The dataset consisted of a total of 705 images. Of these, 80% were used for training, 10% for validation, and the remaining 10% served as the testing subset. The input data for both tooth position identification and alveolar bone level segmentation were consistent across the training, validation, and testing phases. During training, several data augmentation techniques were applied, including horizontal flipping, vertical flipping, Gaussian blurring, and random cropping. Notably, when identifying tooth position, any data augmentation that could alter the tooth’s position should be avoided. The models were evaluated on a separate subset of 70 images. The specific data distribution is shown in Table 1.

**Table 1:** Data distributions of the training set and test set

	Training set	Test set
Number of images	705	70
Number of molars	5332	531
Number of premolars	5481	540
Number of canines	2800	277
Number of incisors	5478	550

The evaluation metrics included the F1 score, intersection over union (IoU), accuracy (PA), sensitivity and specificity. The formulas for these five metrics were as follows:

4 $$F1-score=\frac{{2 \times TP}}{{2 \times TP+FP+FN}}$$

5 $$IoU=\frac{{TP}}{{TP+FN+FP}}$$

6 $$PA=\frac{{TP+TN}}{{TP+FN+FP+TN}}$$

7 $$Sensitivity=\frac{{TP}}{{TP+FN}}$$

8 $$Specificity=\frac{{TN}}{{TN+FP}}$$

where TP (true positives) signifies the number of positive instances accurately identified, demonstrating the model’s proficiency in historically capturing pertinent events. FP (false positives) refers to the instances incorrectly labeled as positive, despite being negative, indicating the model’s commission errors. FNs (false negatives) denote positive instances wrongly classified as negative, underscoring errors of omission that are critical in scenarios where missing positive detections could lead to significant repercussions. The TN (true negatives) represents the number of negative instances correctly identified as such, illustrating the model’s ability to discern nonevents accurately. The F1-score was an equilibrium between precision (the fraction of TP out of all positive predictions) and recall (the fraction of TP out of all actual positives), optimizing the balance between these measures, which is particularly beneficial in imbalanced datasets. The IoU, which is utilized primarily in semantic segmentation evaluation, assesses the congruency between predicted and actual positive regions, accentuating the precision of spatial predictions. The PA computed the ratio of all correct predictions (both TP and TN) within the dataset, providing an overview of the overall model efficacy historically. Sensitivity measures the model’s ability to correctly identify positive instances, which is vital in applications where failing to detect a positive instance is costly. Specificity gauged the ability to accurately identify negative instances, which is crucial in instances where false positives had significant consequences.

The training was conducted over 150 epochs using the AdamW optimizer with a batch size set to 1. The PyTorch 1.12.1 framework facilitated the deep learning process, and all training and testing operations were executed on an NVIDIA GeForce GTX 3090 GPU. The statistical analysis was conducted with MATLAB 2022a software. The correlation coefficient and *p* value were calculated using the ‘corr’ function in MATLAB.

## Results

### Tooth position recognition and tooth semantic segmentation

In the task of tooth position recognition, an impressive IoU of 0.8906 was registered, coupled with an F1-score of 0.9338, positioning it as the pinnacle of precision in predicting tooth placement. The accuracy was slightly tapered in the tooth semantic segmentation task, with an IoU of 0.8465 and an F1-score of 0.9138. However, a juxtaposition of these two tasks in their combined overlay brought forth a subtle decrement in both metrics: the IoU decreased to 0.7889, and the F1-score retreated to 0.8674 (Table 2).

**Table 2:** Performance evaluation for tooth position recognition and tooth semantic segmentation

Task name	IoU	F1-score
Tooth position recognition	0.8906	0.9338
Tooth semantic segmentation	0.8465	0.9138
Overlay of two tasks	0.7889	0.8674
Intrabony portion of the root	0.7837	0.8674
Suprabony portion of the root	0.7121	0.8150
Crown	0.8709	0.9226

IoU, intersection over union

When focusing on the nuanced task of tooth semantic segmentation by category, differences emerge in class-specific accuracies. The tooth crown exhibited the highest precision, registering an IoU of 0.8709 and an F1-score of 0.9226. Following this, the intrabony portion of the root exhibited an IoU of 0.7837 and an F1-score of 0.8674. The suprabony root portion, which may present the greatest segmentation challenge, yielded an IoU of 0.7121 and an F1-score of 0.8150 (Table 2).

### RBL measurements and staging evaluation

**Fig. 4:**
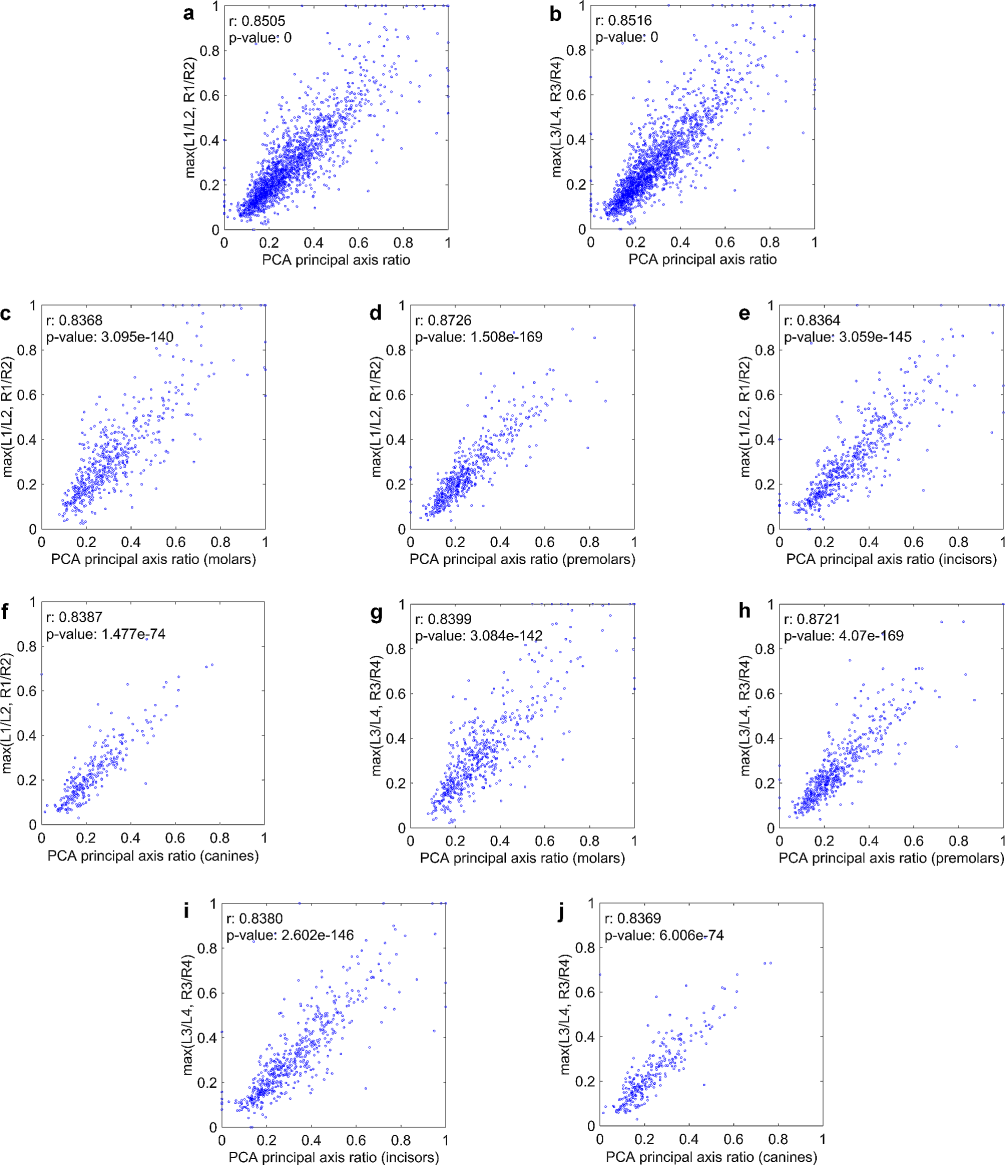
Scatter plot of the PCA principal axis method versus the measurements taken by clinicians. (**a**) and (**b**) scatter plots for all teeth, whereas (**c**)-(**j**) scatter plots for four distinct types of teeth. The x-axis denotes the PCA principal axis ratio, specifically A/B from Fig. 2, and the y-axis represents the measurement values. PCA, principal component analysis

By comparing the RBL values derived from the PCA principal axis to those measured by clinicians in 70 patients in the test set, for a total of 1898 teeth, the DL-based measurements were found to be significantly correlated with the manual measurements (Fig. 4). When the RBL was calculated by measuring the length (L1/L2, R1/R2) and vertical distance (L3/L4, R3/R4), the predicted values showed correlations of 0.8505 and 0.8516, respectively (Fig. 4a and b). The results of the two measurement methods were consistent for different tooth categories (Fig. 4c and j). Notably, premolars displayed the strongest correlation, surpassing 0.87, whereas the other tooth types all consistently achieved coefficients above 0.83. This strong alignment suggested that the RBL values calculated through our approach closely mirrored the manual measurements.

Furthermore, the predictive accuracy of the two clinical methods was different when the severity of alveolar bone resorption was staged according to the maximum RBL permanent teeth of the entire dentition, excluding the third molar (Table 3). Compared with the first measurement method (max (L1/L2, R1/R2) ×100), predictions for Stage I indicated an impressive specificity of 0.9542 and a slight sensitivity of 0.6196. For Stage II, the method showed a sensitivity of 0.7885 and a specificity of 0.7337, indicating potential misclassifications. Stage III was the most consistent, with a balancing sensitivity of 0.7960 and specificity of 0.8980 (Table 3). However, when juxtaposed against the second measurement method (max (L3/L4, R3/R4) ×100), the predictive model slightly improved its sensitivity for Stage I to 0.6451 while preserving comparable specificity. Stage II displayed a modest increase in sensitivity to 0.8046, and the specificity of stage III increased notably to 0.9112, even though the sensitivity marginally decreased (Table 3). Although both methods demonstrated comparable precision, recall, and F1-scores, a closer examination revealed a slight advantage in several aspects of the prediction results for the second method. Moreover, the RBL percentage from the PCA principal axis closely aligned with that from the second measurement method, highlighting its superior values (Fig. 2).

**Table 3:** Evaluation results using the PCA principal axis method. The results include four evaluation metrics: precision, sensitivity, F1 score, and specificity. The ‘Support’ column indicates the sample size for each classification, while the max (L1/L2, R1/R2) and max (L3/L4, R3/R4) represent two measurement methods of reference labels

	Precision	Sensitivity	F1-score	Specificity	Support
Stage I (max (L1/L2, R1/R2))	0.7651	0.6196	0.6847	0.9542	368
Stage I (max (L3/L4, R3/R4))	0.7685	0.6451	0.7014	0.9552	355
Stage II (max (L1/L2, R1/R2))	0.7424	0.7885	0.7648	0.7337	936
Stage II (max (L3/L4, R3/R4))	0.7334	0.8046	0.7674	0.7326	906
Stage III (max (L1/L2, R1/R2))	0.7802	0.7960	0.7880	0.8980	593
Stage III (max (L3/L4, R3/R4))	0.8149	0.7752	0.7945	0.9112	636
Macro avg (max (L1/L2, R1/R2))	0.7625	0.7347	0.7458		1897
Macro avg (max (L3/L4, R3/R4))	0.7722	0.7416	0.7444		1897

## Discussion

The RBL is undeniably a pivotal assessment metric in the expansive domain of periodontal disease and serves as an indispensable tool, offering clinicians invaluable insights into the intricacies and severity of periodontitis. Although intraoral two-dimensional radiographs are the conventional method for assessing RBL, they are often perceived as time-consuming and inherently subjective, leading to variations in assessment among different clinicians. To address these challenges, recent efforts have witnessed a surge in the exploration of automated image processing techniques. Notably, with the advent of DL, a new horizon has emerged for medical image processing. In this context, CNN-based approaches have garnered widespread attention due to their potent feature extraction capabilities. Numerous endeavors employ CNNs to measure precise RBL values or classify RBL levels. Using radiographic findings, such DL models provide a swift and reliable preliminary periodontal diagnosis. Despite the promising performance exhibited by CNNs, their application to panoramic oral images is not devoid of limitations. A primary concern is the restricted receptive field of CNNs, which sometimes struggles when tasked with delineating larger, more complex structures. Unfortunately, downsizing the input image, a common solution, compromises its resolution. This inadvertently diminishes the accuracy of segmentation, a critical component in this assessment. Furthermore, obtaining specific tooth-related RBL values can offer clinicians more nuanced references for periodontal diagnosis—a gap in current methodologies. This study aimed to bridge these gaps by introducing the advanced DL model SegFormer and integrating the PCA methodology to identify the main axis of the tooth structure. This innovative combination is crucial for obtaining accurate RBL values. Broadly, with SegFormer as our foundation, we formulated two essential tasks: tooth position recognition and tooth semantic segmentation.

Recognizing overlapping teeth in panoramic images poses significant challenges. To address this, our study innovated a unique labeling technique utilizing a multi-label loss function to ensure precise recognition of overlapping teeth, circumventing the issue typically seen in segmentation algorithms where a pixel can correspond to only one label.

**Fig. 5:**
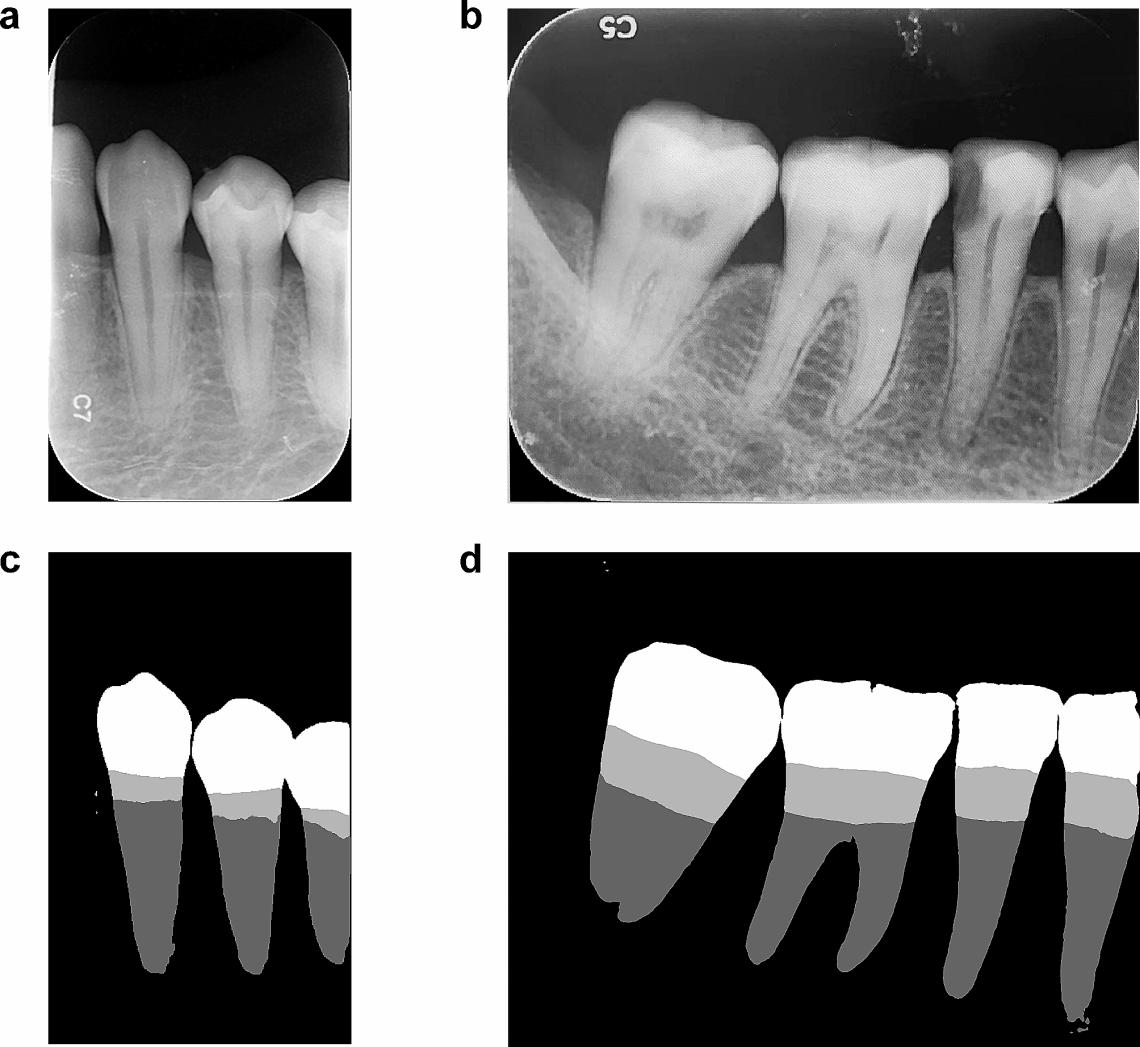
Semantic segmentation of periapical radiographs. (**a**) and (**b**) display two periapical radiographs, while (**c**) and (**d**) represent their respective semantic segmentation results

For tooth semantic segmentation, we identified three key regions: the crown, the suprabony root part, and the intrabony root portion. These areas are quintessential for extracting RBL data. Accurate identification of these segments enables precise RBL evaluation. By amalgamating both the tooth position recognition and dental semantic segmentation tasks, bone-level information for specific teeth can be obtained, allowing for the computation of RBL values based on the lengths the main axis of the tooth structure traverses within these regions. For a set of 70 test images, the SegFormer model yielded average IoU and F1-scores of 0.8906 and 0.9338, respectively, for tooth position recognition. This finding underscores the model’s superior performance in tooth identification even amidst missing or overlapping scenarios. Concurrently, for tooth semantic segmentation, the average IoU and F1 score were 0.8465 and 0.9138, respectively. When both tasks were merged, the average IoU and F1 score reached 0.7889 and 0.8674, respectively. This integration resulted in a slight accuracy reduction, which remained within an acceptable range.

To determine the main axis of the tooth structure, we pioneered the use of the PCA principal axis to emulate the main axis of the tooth structure. The illustrative diagrams provided in the manuscript depict a high degree of congruence between the PCA principal axis and the main axis of the conceptual tooth structure. RBL values derived from PCA on SegFormer’s segmentation results demonstrated correlation coefficients of 0.8505 and 0.8516 with two measurement techniques, indicating a high correlation with clinician-measured outcomes. According to the tooth position-specific statistics, the anterior molars had the highest correlation coefficient, exceeding 0.87, whereas the other tooth types also had a correlation coefficient greater than 0.83. Therefore, the obtained RBL value has high reliability compared to the measurements made by clinicians. Based on the 2018 staging standards, we compared the staging accuracy of the proposed method against that of two clinical measurement techniques, emphasizing the reliable staging accuracy of this method. The proposed approach, which encompasses tooth position recognition, tooth semantic segmentation, and PCA-based RBL computation, offers several advantages over existing DL paradigms. These include deriving precise RBL values for specific teeth, higher automation levels, fewer requisite steps with the capability to handle panoramic images, heightened efficiency, and greater mathematical interpretability and reliability in diagnosis due to the PCA principal axis determination. However, certain limitations still exist. Even though SegFormer has fewer parameters and data augmentation was employed during training, more images are essential for further enhancing its performance and efficiency. Currently, on our hardware, the inference speed for tooth position recognition and tooth semantic segmentation was 1.54 s, which means that it takes 1.54 s to process a single panoramic image. Additionally, when a tooth’s shape is elongated along the main axis direction of the tooth structure and shortened in the direction perpendicular to it, the principal axis of the PCA might represent a direction perpendicular to the main axis of the tooth structure, although such scenarios are exceedingly rare. Notably, this method aids clinicians by providing RBL reference values for treatment planning and does not diagnose periodontitis at different stages. Moreover, segmenting features such as crowns can assist in diagnosing other dental conditions, highlighting the versatility of these methods. Our model also demonstrated notable transferability capabilities. Although SegFormer was originally trained on panoramic images, it proved effective in semantic segmentation tasks on periapical radiographs, as illustrated in Fig. 5. Although we ceased utilizing the tooth position recognition model, the semantic segmentation outcomes continued to offer valuable insights for clinicians. This finding exemplified the robust generalization capacity inherent to deep learning models, which allowed them to seamlessly adjust to diverse data types. To extract RBLs for individual teeth in periapical radiographs, one simply needs to tweak the recognition target of the tooth position model to differentiate individual teeth. In essence, our approach showcased flexibility, necessitating only slight modifications to meet the unique demands of each specific task.

## Data Availability

No datasets were generated or analysed during the current study.
